# Draft Genome Sequence of *Flavobacterium psychrophilum* Strain KTEN-1510 with Genotype A/G-C, Isolated from an Ayu (*Plecoglossus altivelis altivelis*) in the Kagami River, Kochi, Japan

**DOI:** 10.1128/genomeA.01762-15

**Published:** 2016-02-18

**Authors:** Masato Shimizu, Hikaru Goda, Kenichi Yamasaki, Syun-ichirou Oshima, Kouhei Ohnishi, Yasuo Osaki, Shigehiko Kataoka, Masayuki Imajoh

**Affiliations:** a Department of Aquaculture, Fish Disease Laboratory, Kochi University, Nankoku, Kochi, Japan; b Graduate School of Kuroshio Science, Kochi University, Nankoku, Kochi, Japan; c Research Institute of Molecular Genetics, Kochi University, Nankoku, Kochi, Japan; d Kagamigawa Fisheries Cooperative Association, Kochi, Kochi, Japan

## Abstract

In this paper, we describe the draft genome sequence of *Flavobacterium psychrophilum* strain KTEN-1510, with genotype A/G-C. This strain was isolated in October 2015 from the gills of an ayu (*Plecoglossus altivelis altivelis*) in the upper Kagami River in central Kochi Prefecture on Shikoku Island, Japan.

## GENOME ANNOUNCEMENT

Ayu (*Plecoglossus altivelis altivelis*) is a popular angling fish in Japan. A large number of juvenile ayu reared in hatcheries or collected from Lake Biwa, local rivers, and marine coasts are released annually in fishing areas to enhance ayu stocks. *Flavobacterium psychrophilum*, a member of the family *Flavobacteriaceae* (1) of the order *Flavobacteriales* (2) within the class *Flavobacteriia* (3), is the etiological agent of bacterial cold-water disease (BCWD) in ayu (4). BCWD has affected ayu populations in lakes and rivers across Japan since the mid-1990s, following the release of infected individuals, which has caused a significant commercial loss to the fishing industry (5). Outbreaks of BCWD most frequently occur from May to July at water temperatures of 14 to 21°C, as reported by the Ministry of Agriculture, Forestry and Fisheries (http://www.maff.go.jp/j/syouan/suisan/suisan_yobo/ayu_reisui/).

The Kagami River is located in the central Kochi Prefecture on Shikoku Island in Japan. The river is 31 km long, with a drainage basin area of 170 km^2^. The Kagami Dam, in the middle of the river, was built in 1967. In late June 2014, there was a mass die-off of ayu in the upper reaches of the dam, and *F. psychrophilum* is thought to have been responsible (data not shown). In this paper, we describe the draft genome sequence of *F. psychrophilum* strain KTEN-1510, isolated in October 2015 from the gills of an ayu caught in the upper reaches of the dam using the Japanese fishing method known as Tomozuri.

The genomic DNA of KTEN-1510 was extracted, purified, and sequenced according to our previously used method (6). Genome assembly was performed using the GS *de novo* Assembler version 2.9 software (Roche). The assembly of strain KTEN-1510 consisted of 182 contigs (>500 bp) totaling 2,705,210 bp, with a G+C content of 32.5%. We annotated the sequence using the Microbial Genome Annotation Pipeline (MiGAP) version 2.19 (7). A total of 2,608 protein-coding sequences (CDSs) were predicted, and we identified at least 38 tRNA genes and at least four rRNA operons. We used the Rapid Annotations in Subsystems Technology (RAST) server version 2.0 (8) for subsystem descriptions. According to the RAST server analysis, there were 320 subsystems, but subsystem features for photosynthesis, motility, and chemotaxis were not found. Yoshiura et al. (9) classified 87 *F. psychrophilum* isolates into two genotypes, A and B, based on the PCR-restriction fragment length polymorphism (PCR-RFLP) assay of the peptidyl-prolyl *cis*-*trans*-isomerase C (*PPIC*) gene. Type A was detected only in isolates from ayu. Fujiwara-Nagata et al. (10) identified four genotypes (G-C, A-T, A-C, and G-T) among 232 isolates using single-nucleotide polymorphism (SNP) analysis of the DNA gyrase subunit A (*gyrA*) gene. Type G-C showed strong pathogenicity to ayu, whereas the other three were not or only weakly pathogenic. Both PCR-RFLP- and SNP-based genotyping identified strain KTEN-1510 as type A/G-C (data not shown). The present whole-genome analysis of the virulent strain of BCWD increases our understanding of the mechanisms underlying the pathogenicity of *F. psychrophilum*.

### Nucleotide sequence accession numbers.

The draft genome sequence of strain KTEN-1510 has been deposited in GenBank under accession no. BCNG00000000. The version described in this paper is the first version, BCNG01000000.
